# The Effects of Acute Beetroot Juice Intake on Glycemic and Blood Pressure Responses When Controlling for Medication in Individuals with Type 2 Diabetes: A Pilot Study

**DOI:** 10.3390/nu16162636

**Published:** 2024-08-10

**Authors:** Andrew P. Tyler, Braxton A. Linder, Karina Ricart, Christian E. Behrens, Fernando Ovalle, Rakesh P. Patel, Gordon Fisher

**Affiliations:** 1 Department of Human Studies, School of Education and Human Sciences, University of Alabama at Birmingham, Birmingham, AL 35294, USA; aptyler@uabmc.edu; 2 Department of Kinesiology, School of Public Health, Indiana University, Bloomington, IN 47405, USA; balinder@iu.edu; 3 Center for Free Radical Biology, Department of Pathology, University of Alabama at Birmingham, Birmingham, AL 35294, USA; karinaricart@uabmc.edu (K.R.); rakeshpatel@uabmc.edu (R.P.P.); 4 Bayer Consumer Health Care, Whippany, NJ 07981, USA; christian.behrens@bayer.com; 5 Division of Endocrinology, Diabetes and Metabolism, Department of Medicine, University of Alabama at Birmingham, Birmingham, AL 35294, USA; fovalle@uabmc.edu

**Keywords:** cardiometabolic health, type 2 diabetes, beetroot juice, dietary nitrate, vascular health, insulin resistance, glucose uptake

## Abstract

Physical inactivity and poor dietary choices contribute to the rise in cardiometabolic diseases in the United States. It remains critical to identify strategies that may mitigate the negative impact of these behaviors. Several studies have shown that the consumption of dietary inorganic nitrate may improve vascular health and glucose regulation in animal models and some human studies. However, the improvements in glucose regulation have yet to be corroborated in humans with type 2 diabetes (T2D). Therefore, the purpose of this study was to assess the acute effects of beetroot juice (BRJ) on glycemic and hemodynamic responses in individuals with T2D while controlling for medication. Seven participants with a clinical diagnosis of T2D were recruited into this study and were temporarily removed from blood pressure- and glucose-lowering medications. Hemodynamic measurements (pulsewave velocity) and an oral glucose tolerance test (glycemic response) were measured following consumption of either BRJ or a denitrolized placebo. Saliva and blood samples were collected at baseline and two and four hours post supplementation to measure changes in nitrate and nitrite concentrations. We detected significant improvements in total plasma glucose exposure (*p* = 0.022) and the SVR change score (*p* = 0.009) in the BRJ condition. This study demonstrated that BRJ consumption can improve oral glucose tolerance in individuals with T2D while controlling for medication; however, future larger-cohort randomized controlled trials are needed to confirm if BRJ is a viable treatment for glucose control in individuals with T2D.

## 1. Introduction

Cardiometabolic diseases, such as type 2 diabetes (T2D) and hypertension, continue to rise within the United States and other parts of the Western world [1,2]. T2D and hypertension have both been characterized by increased oxidative stress, atherosclerosis, endothelial dysfunction and insulin resistance [3,4]. Thus, the pathophysiology and etiology of these diseases are multifaceted and likely develop from a number of overlapping mechanisms. Nitric oxide (NO) availability has been shown to be beneficial to cardiovascular health, by regulating vascular functioning and improving blood flow [5,6]. Additionally, NO has also been demonstrated to have therapeutic effects on glucose tolerance and glycemic control [7,8]. NO can be produced endogenously through the enzymatic reaction of endothelial nitric oxide synthase (eNOS) to oxidize the amino acid L-arginine into NO [7,9]. Within healthy ranges, insulin acts to increase NO production to improve glucose uptake within the microvasculature around skeletal muscle [10]. However, when insulin resistance develops, phosphatidylinositol 3 kinase-nitric oxide (P13K-NO) activity decreases [10], resulting in impaired downstream eNOS activity characteristic of endothelial dysfunction, and leads to diminished NO bioavailability [11,12,13].

However, it is now well known that the consumption of foods containing dietary inorganic nitrate (diNO3), such as dark leafy greens and root vegetables, can exogenously enhance NO bioavailability independent of eNOS [5,14]. This pathway utilizes nitrate reductase-containing bacteria in the oral microbiome to reduce nitrate into nitrite before being absorbed into the systemic circulation, where it acts to increase local NO bioavailability [3,7,15,16,17]. The ingestion of dietary sources rich in diNO3 can increase nitrate levels nine-fold and nitrite levels six-fold [18]. Preclinical data suggest that chronic diNO3 may reduce blood pressure, improve glycemic control, and improve insulin responses [19,20]. Specifically, both studies observed that ~8 weeks of nitrate supplementation improved markers of cardiometabolic health in rats with diabetes when compared to untreated diabetic rats. Interestingly, the difference observed in systolic blood pressure between the diabetic and control rats was ameliorated in the diabetic rats given nitrate [19].

In humans, significant reductions in basal plasma glucose have been observed after a single bolus of diNO_3_ [21], though effects on glucose tolerance, insulin resistance, and blood pressure have not been observed in patient populations with T2D diagnosis [21,22]. One possible explanation for why no effect of diNO_3_ has been observed in this population is that participants were not removed from blood pressure-lowering and glucose-lowering medications during the trials. Specifically, the mechanisms of action of such pharmaceuticals, specifically metformin, also utilize the NO pathways of the body [23,24]. Thus, it is possible that the lack of effect on cardiometabolic responses from the diNO_3_ supplements in these studies was because of participants’ current medication already mediating the intended effects of the diNO_3_ supplements. Therefore, the purpose of this pilot trial was to deliver proof of concept that (determine whether) diNO_3_ supplementation, in the form of beetroot juice (BRJ), may improve cardiometabolic responses in humans with T2D without the influence of metformin-dependent blood pressure-lowering and glucose-lowering effects.

## 2. Materials and Methods

### 2.1. Study Design and Participants

Seven participants (two male/five female; four Black/three White; 57 ± 8 years; resting BP = 133 ± 18/77 ± 12; intake HbA1c = 6.4 ± 0.7; Table 1) were recruited from the University of Alabama at Birmingham’s Diabetes and Endocrinology Clinic in a randomized crossover design using two beetroot juice (BRJ) conditions: (1) NO_3_^−^-rich beetroot juice (BRJ; approximately 6.4 mmol of NO_3_^−^ per 70 mL; Beet it; James White Drinks, Ipswich, UK), or (2) NO_3_^−^-depleted beetroot juice (PLA; approximately 0.04 mmol of NO_3_^−^ per 70 mL; Beet it; James White Drinks, Ipswich, UK). Inclusion criteria for the study included the following: age between 40 and 65 years old, clinical diagnosis of T2D from a primary care physician or endocrinologist, HbA1c ≤ 9% at the screening visit, blood pressure < 140/90, and willing to stop taking diabetes medication for up to four weeks and blood pressure medication for up to three weeks (this was the maximum time period participants would be off their medication; the time period varied based on participants availability to schedule study visits). Exclusionary criteria included the following: taking more than two medications for either diabetes or blood pressure management, taking either pioglitazone or rosiglitazone for diabetes management, current antioxidant supplementation, or unwillingness to refrain from mouthwash usage or planned physical activity for the duration of the study. All protocols were reviewed and approved by the UAB IRB.

Participants were scheduled for three study visits. Informed consent was given on the first visit after HbA1c, blood pressure, height and weight were measured. Participants were instructed to stop taking their diabetes medications for a minimum of two weeks and blood pressure medications for a minimum of one week prior to the second visit. Visits two and three were scheduled as soon as participants were removed from their diabetes medication for a minimum of 2 weeks and blood pressure medication for at least 1 week. Visits two and three were separated by at least 72 h to ensure a sufficient washout period. Participants were randomized in a double-blind crossover design for visits two and three. Testing visits for both conditions were identical and started between 8:00 a.m. and 9:00 a.m. following an overnight fast for at least 12 h. Participants were instructed to rest for 10 min upon arrival. After the 10 min wait, blood pressure was measured and pulsewave analysis was conducted at three different time points followed by a blood draw and saliva collection. Upon completion of baseline measurements, the participant was given 140 mL of BRJ or PLA. Participants remained in the Clinical Research Unit (CRU) for two hours, and then baseline measurements were repeated. Participants were then administered an oral glucose-tolerance test for ~2 h. Upon completion of the OGTT, final blood and saliva samples were collected.

### 2.2. Nitrate and Nitrite Analysis

Blood was drawn into two vaccutubes, one containing EDTA and the other containing citrate. The citrate tube was spun at 5000 Gs for three minutes to separate the blood. Supernatant was separated and placed into 100 µL aliquots, then flash-frozen in liquid nitrogen and stored at −80 °C until analysis. The EDTA tubes sat for at least ten minutes, then spun at 5000 Gs for ten minutes to separate the blood. Supernatant was placed into 100 µL aliquots and stored at −80 °C until analysis. Saliva samples were collected in tandem with plasma samples. Then, 1.5 mL of saliva was spun at 3000 Gs for three to five minutes, or until separation was visible. Two 100 µL aliquots of salivary supernatant were collected, flash-frozen in liquid nitrogen, and stored at −80 °C until analysis. For plasma and saliva, nitrate and nitrite samples were thawed, methanol was added to plasma and saliva (2:1 ratio), the vortex was mixed, and the supernatant was collected. Nitrite and nitrate samples were then measured with methanolic extracts using HPLC coupled with the Griess reaction using an ENO-30 (EiCom, Kyoto, Japan). Nitrate and nitrite levels were calculated by comparison to standard curves generated daily [25].

### 2.3. Vascular Hemodynamics

Hemodynamic variables were measured using the HDI/Pulse Wave TM CR-2000 (Hypertension Diagnostics, Eagan, MN, USA) for the first three participants. Due to the COVID-19 pandemic, access to research facilities was restricted, and the HDI/PulseWave TM CR-2000 was misplaced. The remaining four participants had their vascular hemodynamics assessed via SphygmoCor Xcel^®^ (AtCor Medical, Naperville, IL, USA). Participants rested for 10 min prior to data collection. Systolic blood pressure (SBP), diastolic blood pressure (DBP), pulse rate (PR), augmentation index (AIX), augmented pressure (AP), and augmentation index 75 (AIX75) were all collected. Measurements were collected using a standard brachial cuff on the left arm of each participant. Data were collected in triplicate at baseline (PRE) and 2.5 h after (POST) the ingestion of both supplements.

### 2.4. Oral Glucose Tolerance Test (OGTT)

Insulin sensitivity and glycemic responses were assessed using a two-hour oral glucose-tolerance test (OGTT). Prior to the start of the OGTT, a catheter was placed in the antecubital space of the right arm. Two blood samples were taken 10 and 5 min before the ingestion of a 75 g dextrose solution at time 0 to assess fasting glucose measures and to provide baseline measures. After the ingestion of the dextrose load, blood samples were collected at 10, 20, 30, 60, 90, and 120 min. Blood was collected to measure serum glucose, insulin, and C-peptide concentrations. Serum was stored in a −80 °C freezer until analysis. OGTT serum was analyzed in the Metabolism Core on the UAB campus. Using data from the OGTT analysis, insulin sensitivity was measured using the Matsuda index and Quantitative Insulin Sensitivity Check Index (QUICKI). Insulin resistance was assessed using the Homeostasis Model of Assessment of Insulin Resistance (HOMA-IR).

### 2.5. Statistical Analysis

All data were assessed for normality using the Shapiro–Wilks test. Normally distributed data are presented as means ± SD, and non-normally distributed data are expressed as median (IQR) change scores. To determine nitrite and nitrate responses in the plasma and saliva, we used two-way repeated measures ANOVA (Condition × Time); none of these variables were normally distributed and therefore a log-transformation was applied to the data (plasma nitrite, plasma nitrate, salivary nitrite, salivary nitrate) [26]. Similarly, we used two-way repeated measures ANOVA (Condition × Time) to assess hemodynamic responses (SBP, DBP, MAP, SVR, AP, AIX75) and glycemic responses to the OGTT (glucose, insulin, and C-peptide). Additionally, calculated variables included area under the curve (AUC), calculated using trapezoidal summation on all glycemic responses to the OGTT. The Matsuda index, HOMA-IR, and QUICKI were all calculated from the OGTT [27], and change scores were calculated for all hemodynamic responses. The calculations are as follows:

*Matsuda* = 10,000/√[*fasting glucose* (mmol/L) × *fasting insulin* (pmol/L] × [*mean OGTT glucose* (mmol/L) × *mean OGTT insulin* (pmol/L)]

*HOMA-IR* = [(*fasting insulin* (uIU/mL) × *fasting glucose* (mmol/L))/405

*QUICKI* = 1/(*log*_10_(*fasting glucose* (mg/dL) + *log*_10_(*fasting insulin* (uIU/mL))

*Change score* = (*post* − *pre*)/*pre*

Paired two-tailed *t* tests and the nonparametric Wilcoxon ranked test were used to detect differences in calculated variables. α was set a priori to <0.05. Statistical analyses were performed by using the Statistical Package for the Social Sciences (SPSS) version 26.0.0 (IBM, Armonk, NY, USA) and jamovi 2.3.18 [28]. Figures were generated in GraphPad Prism 10.2.1.

## 3. Results

### 3.1. Descriptive Statistics

Participants were adults that were removed from blood pressure-lowering and blood glucose-lowering medication for the duration of the study. Prescribed type 2 diabetes medications included metformin, semaglutide and empagliflozin. Descriptive data are displayed in Table 1.

### 3.2. Nitrate and Nitrite Responses

**Figure 1:**
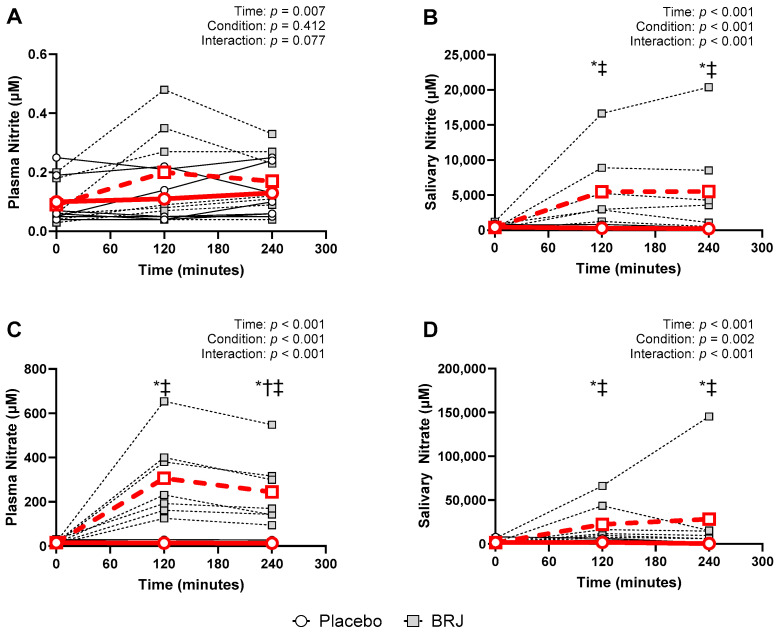
Circulating and salivary nitrite and nitrate data (Individual data shown as legend dictates; average data shown in bold red; the dashed red line represents the BRJ condition, and the solid red line represents the placebo condition). *—indicates significant differences between BRJ and PLA; †—indicates a significant difference from the 0 h timepoint for PLA condition; ‡—indicates a significant difference from the 0 h timepoint for the BRJ condition. (A) 2-h time course of plasma nitrite after supplementation. (B) 2-h time course of salivary nitrite after supplementation. (C) 2-h time course of plasma nitrate after supplementation. (D) 2-h time course of salivary nitrate after supplementation.

All nitrate and nitrite responses violated the Shapiro–Wilks test of normality (*p* < 0.05) at at least at one timepoint included in the analysis; therefore, all data were log-transformed to normalize distribution [26]. We observed the significant effects of time across plasma and salivary nitrate and nitrite responses (*ps* < 0.007). There was not a significant effect of condition on plasma nitrite (*p* = 0.412), nor a significant interaction (*p* = 0.077); however, we did observe significant effects of condition (*ps* < 0.002) and significant interactions (*ps* < 0.001) across plasma nitrate, salivary nitrate, and salivary nitrite. Bonferroni post hoc tests were conducted to examine pairwise differences (Figure 1).

### 3.3. Hemodynamic Responses

**Figure 2:**
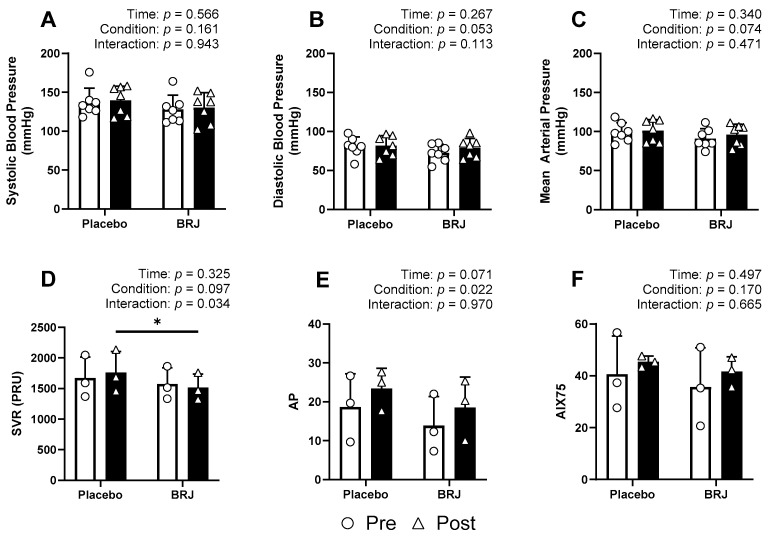
Hemodynamic data compared by timepoint (pre = white bars; circles; post = black bars; triangles). (**A**) Systolic blood pressure responses. (**B**) Diastolic blood pressure responses. (**C**) Mean arterial pressure responses. (**D**) Systemic vascular resistance responses. (**E**) Augmentation pressure responses (**F**) Responses of augmentation index normalized to a heart rate of 75 beats per min. * denotes a significant difference (*p* < 0.05).

**Figure 3:**
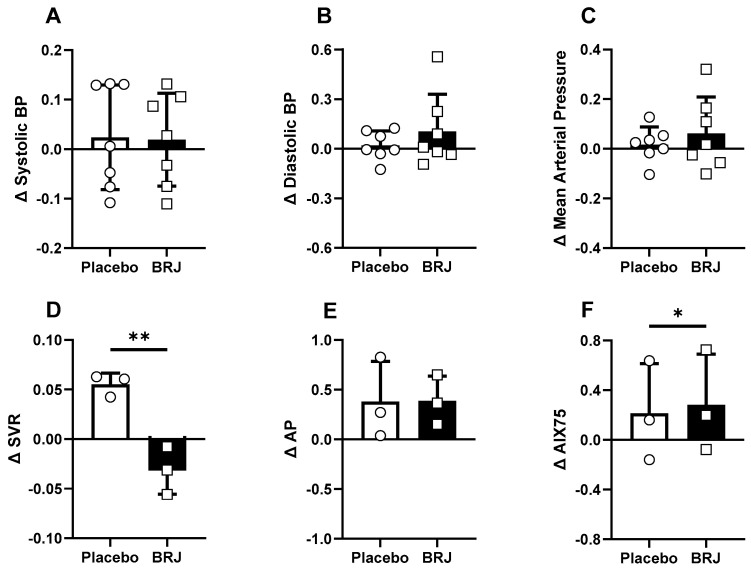
Normalized hemodynamic change scores ‘(post − pre) / pre’ compared between condition. (**A**) Systolic blood pressure change scores. (**B**) Diastolic blood pressure change scores. (**C**) Mean arterial pressure change scores. (**D**) Systemic vascular resistance change scores. (**E**) Augmentation pressure change scores (**F**) Change scores of augmentation index normalized to a heart rate of 75 beats per min. * denotes a significant difference (*p* < 0.05). ** denotes a significant difference (*p* < 0.01).

None of our hemodynamic variables violated normality at any time point (Shapiro–Wilk; *p* > 0.05). We observed no significant effects of time, condition, or interaction on SBP *(ps* > 0.161), DBP *(ps* > 0.053), MAP (*ps* > 0.074), or AIX75 (*ps* > 0.170). We did observe significant effects of condition on AP (*p* = 0.022) and AIX (*p* = 0.049), and a significant interaction in SVR (*p* = 0.034). Bonferroni post hoc tests were conducted to examine pairwise differences (Figure 2). When comparing the normalized change scores, BRJ was shown to significantly reduce SVR (*p* = 0.009, d = 6.04) and increase AIX75 (*p* = 0.048, d = 2.53). Significance was not detected from any other change score comparison (*ps* > 0.366) Figure 3.

### 3.4. Glycemic Responses to the OGTT

**Figure 4:**
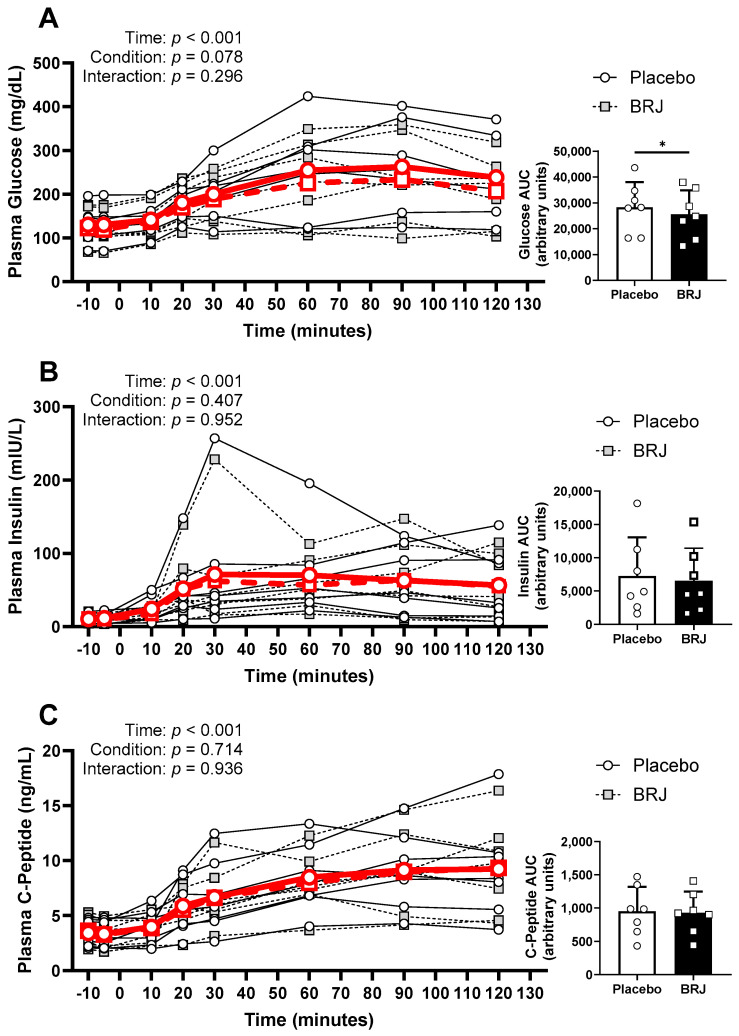
OGTT response data (Individual data shown as legend dictates; average data shown in bold red). (**A**) OGTT curve, representing time specific glucose concentration, and individual AUC data (*right*) representing total plasma glucose exposure. (**B**) OGTT curve, representing time specific insulin concentration, and individual AUC data (*right*) representing total plasma insulin exposure. (**C**) OGTT curve, representing time specific c-peptide concentration, and individual AUC data (*right*) representing total plasma c-peptide concentration. * denotes a significant difference (*p* < 0.05).

**Figure 5:**
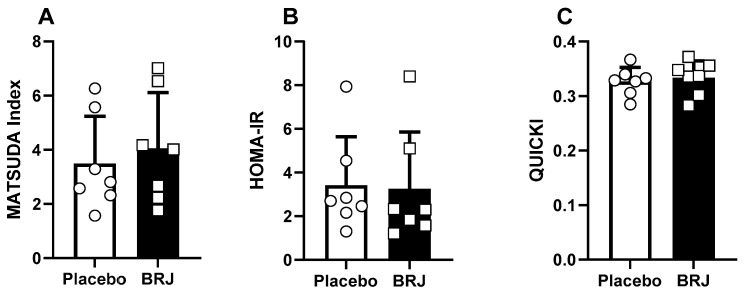
Glycemic control data calculated from the 2-h OGTT. (**A**) represents the MATSUDA calculation, (**B**) represent the HOMA-IR calculation, and (**C**) represents the QUICKI calculation.

Blood glucose and C-peptide concentrations were normally distributed at all time points for both conditions, while insulin violated normality and was therefore log-transformed to normalize distribution. There was an effect of time on blood glucose (*p* < 0.001), but the condition (*p* = 0.429) or interaction (*p* = 0.173) had no effect. Similarly, there was an effect of time on insulin (*p* < 0.001), and no effect produced by condition (*p* = 0.407) or interaction (*p* = 0.952). Again, there was an effect of time on C-peptide (*p* < 0.001) and no effect of condition (*p* = 0.714) or interaction (*p* = 0.936) (Figure 4). However, there was a significant decrease in total glucose exposure (AUC) in the BRJ condition (*p* = 0.022, d = 1.16) but not insulin (*p* = 0.123) or C-peptide (*p* = 0.492). Additionally, there were no differences detected between conditions using Matsuda (*p* = 0.187), HOMA-IR (*p* = 0.873), or QUICKI (*p* = 0.491) (Figure 5).

## 4. Conclusions

In this pilot trial, we observed that an acute dose of BRJ lowered total plasma glucose exposure, reduced SVR, and increased NO bioavailability in participants diagnosed with T2D. Specifically, this study is the first to assess the effects of diNO3 when participants are removed from both hypertension and anti-diabetic medications. Since this is a pilot trial with a relatively low sample size, the results presented should be interpreted with nuance; however, we have generated useful data interrogating the effects of BRJ on ameliorating the stresses associated with cardiometabolic diseases.

As anticipated, we observed increases in plasma nitrate, salivary nitrate, and salivary nitrite [18]. Plasma nitrite increases were not statistically significant (although trends towards significance (*p* = 0.07) were noted) with the acute BRJ supplement. This likely reflects the fact that changes in plasma nitrite are lower in magnitude compared to nitrate—a consequence of multiple, relatively rapid, pathways that lead to nitrite oxidation or reduction [3,7,15,16,17]. Within circulation, there are many enzymatic and nonenzymatic processes that can reduce nitrite to NO [29,30], in which local acidity and oxygen saturation play major roles.

Though the causality of insulin resistance and local acidosis is still unknown, it is known that there is a strong relation between the two dysregulated states [31]. Therefore, what we may have observed is the reduction of nitrite to NO near the resistance vessels within the periphery, which could explain both the reduction of SVR and improved glucose uptake despite no effects on insulin. As mentioned earlier, insulin typically acts to increase eNOS activity to improve perfusion and glucose uptake in the periphery [10]. Though there is still work to be conducted to elucidate whether this was mediated by the diNO_3_ within the BRJ or one of its other active constituents, we are the first, to our knowledge, to observe decreases in SVR and total glucose exposure in response to BRJ in a population with insulin resistance. Furthermore, while we did not see statistically significant improvements for Matsuda, QUICKI, or HOMA-IR from the OGTT test, it is worth noting that this is likely due to the limited sample size in this pilot study, given we saw improvements for each of these variables in six out of seven of the study participants. This is important as previous work has not demonstrated significant effects of BRJ in glucose tolerance [21,22], which we hypothesized was due to the participants remaining on blood pressure-lowering and blood glucose-lowering medications. Additionally, it may be worth incorporating assessments of SVR and microvascular function with these supplements in the future, especially in light of the microvascular dysregulation common with metabolic disorders, as opposed to only measures of conduit artery function such as BP and flow-mediated dilation. Although we can only speculate as to why there were no measurable improvements for SBP, DBP, or arterial stiffness in our study, it is possible that the effects of diNO_3_ may be mediated within the microvasculature, as SVR is more reflective of resistance within the smaller resistance arterioles. Thus, there could be improvements within the microvasculature leading to improvements in glucose clearance without significant effects on blood pressure.

We also only used an acute high dose of BRJ. Something to consider is the potential of short-term, long-term, and chronic studies to understand whether repeated exposure to NO intermediaries may improve cardiovascular outcomes associated with long-term endothelial dysfunction as a result of impaired eNOS activity. In 2019, a short-term BRJ supplementation study was conducted that used participants of a similar age to our cohort; these participants were otherwise generally healthy, and this trial demonstrated that 2 weeks of BRJ was enough to observe decreases in SBP and DBP in healthy older adults [32]. Furthermore, in healthy young adults, it has been demonstrated that 15 days of BRJ supplementation was enough to reduce resting blood pressures and blood pressures during submaximal, dynamic exercise [33], which indicates a lower risk of cardiovascular disease-related events [34]. These effects in healthy populations highlight the need to conduct future studies investigating optimal dosing periods for clinical populations.

Another factor that may impact the efficacy of BRJ in helping cardiometabolic responses is the fact that we were also predominantly working with participants with concomitant obesity. While comorbid with T2D, obesity is also associated with chronic, low-grade inflammation that further accelerates and exacerbates the progression of T2D [35]. Prior work by our group has demonstrated that even an acute high dose of BRJ was enough to reduce submaximal VO_2_ and improve time to exhaustion in an untrained, obese population [36]. Though an acute dose of BRJ was not enough for us to detect clinical changes in vascular function, these data suggest that an acute dose of BRJ may still be useful in improving exercise tolerance in our population. Though causality between obesity and T2D is still contested, it may be worth implementing BRJ supplementation or dietary practices that increase NO bioavailability, while also implementing lifestyle modifications as a means of slowing the progression, and potentially reversing, some of these chronic diseases.

One thing we have yet to investigate is the effect of the other constituent parts of BRJ on these cardiometabolic measures within our population. Specifically, metabolic diseases, like T2D, are heavily influenced by oxidative stress [37]. Not only has excessive oxidative stress been implicated in metabolic diseases, but it has also been identified as a major risk factor for the development of vascular diseases such as atherosclerosis [38]. While BRJ is an effective means of consuming diNO_3_, BRJ is also rich in antioxidants [39], which serve as a means of neutralizing oxidative stress. Implementing a design in which an NO_3_-depleted BRJ supplement is used would enhance our understanding of how the different components of BRJ interact individually or may be synergistically cardioprotective.

We acknowledge that our pilot study has its limitations, in particular the sample size. While this is a pilot study, we find it encouraging that with such a limited sample we were still able to detect significance within our analyses. However, to our knowledge, no one has yet assessed the effects of BRJ on cardiometabolic measures in people diagnosed with T2D that have been removed from their medications for the duration of the study. Therefore, we hope this pilot trial provides proof of concept and the ability to calculate the statistical power needed to investigate these questions in this population. Second, we would like to acknowledge the impact of the COVID-19 pandemic on our data collection—specifically, the use of two different devices to assess vascular health. Due to the shutdown and reprioritization of clinical research spaces, our HDI/Pulse Wave TM CR-2000 was misplaced. At that time, three participants had already completed both trials, and by the time data collection was able to continue, we could not order another HDI/Pulse Wave TM CR-2000. Thus, the SphygmoCor was used.

## Figures and Tables

**Table 1:** Participant demographics.

Variable	Mean ± SD
Sex	5F/2M
Race	4B/3W
Age	57 ± 8
Height (cm)	166 ± 15
Body Mass (kg)	99.3 ± 29.1
BMI	36.9 ± 13.5
HbA1c	6.4 ± 0.7
SBP (mmHg)	133 ± 18
DBP (mmHg)	77 ± 12
MAP (mmHg)	95 ±13

Descriptive data for study participants: Body Mass Index (BMI), Glycated Hemoglobin A1C (HbA1c), Systolic Blood Pressure (SBP), Diastolic Blood Pressure (DBP), and Mean Arterial Pressure (MAP).

## Data Availability

The data in this study are available on request due to privacy.
